# Evaluation of treatment regimens and long-term clinical outcomes in patients with isoniazid-resistant pulmonary tuberculosis: a 5-year follow-up

**DOI:** 10.55730/1300-0144.5639

**Published:** 2023-01-04

**Authors:** Ayşe Feyza ASLAN, Mediha Gönenç ORTAKÖYLÜ, Belma Akbaba BAĞCI, Sezer TOPRAK

**Affiliations:** 1 Department of Pulmonary Medicine, University of Health Sciences, Yedikule Chest Diseases and Thoracic Surgery Training and Research Hospital, İstanbul, Turkey; 2 Department of Microbiology, University of Health Sciences, Yedikule Chest Diseases and Thoracic Surgery Training and Research Hospital, İstanbul, Turkey

**Keywords:** Fluoroquinolone, tuberculosis, treatment, isoniazid resistance

## Abstract

**Background/aim:**

Considering its early bactericidal activity, isoniazid (H) is an important first-line agent in tuberculosis (TB) treatment. The aim of this study was to evaluate the treatment regimens and results of H-resistant pulmonary TB patients.

**Materials and methods:**

We retrospectively evaluated treatment regimens and results of 188 H-resistant pulmonary TB patients who were treated in our center between January 2015 and December 2017. Treatment regimens applied were noted and treatment outcomes were recorded. The long-term results were evaluated.

**Results:**

Totally 174 (92.6%) of 188 patients with H-resistant pulmonary TB achieved treatment success. Ninety-seven patients (51.6%) were cured and 77 patients (41.0%) completed treatment. Five patients (2.7%) had treatment failure. Four patients (2.1%) having treatment success relapsed during one-year follow-up. Eighteen patients (9.6%) had unfavorable outcomes, including treatment failure in five (2.7%), death in nine (4.8%), and relapse in four patients (2.1%). The treatment success rate was found to be statistically higher in group 1 (9-month regimen 2HREZ/7HRE) compared with those in group 2 (9HREZ) (97.4% vs. 85.9%; p = 0.010). Group 3 (HREZFQ) and group 1 had statistically significant favorable outcomes, compared to group 2 (group 2 vs. group 3, p = 0.048; group 1 vs. group 2, p = 0.022). Interestingly, no relapse and acquired rifampicin resistance in patients occurred who received an FQ-containing regimen.

**Conclusion:**

Our study results show higher treatment success and positive results with the treatment regimen containing FQ and that treatment with HREZ for nine months is associated with a lower treatment success rate.

## 1. Introduction

Around 10 million individuals worldwide are diagnosed with tuberculosis (TB) disease every year. Tuberculosis is one of the 10 most important causes of death, and Mycobacterium tuberculosis (M.tb), a single infectious agent, is the main cause. Although the disease can affect all age groups in all settings, the majority of patients (about 90%) who develop TB are adults. The male: female ratio is 2:1 and nearly 90% of cases each year occur in 30 countries with a high TB burden. An estimated 1.7 billion individuals globally are infected with M. tb and at risk of developing active TB^1^

Isoniazid-resistant TB (Hr-TB) refers to strains of M.tb that are Hr and susceptible to rifampicin (R) as confirmed in vitro^2^. Isoniazid (H) is one of the most widely used first-line drugs in the treatment of active TB and latent TB infection (LTBI), with high bactericidal activity and a good safety profile. The emergence of Hr-TB strains endangers and reduces the effectiveness of TB treatment^1^. It is estimated that approximately 8% of TB patients have R-sensitive, Hr-TB worldwide^2^.

In M.tb, drug resistance develops through spontaneous genetic mutations. Therefore, the development of acquired drug resistance usually occurs, when there is a large bacterial population, such as in cavitary pulmonary TB, or when an inadequate combination or dosage of drugs is prescribed [1]. Rarely, malabsorption of TB drugs may explain acquired resistance [2]. Risk factors for developing H resistance have been reported, and most studies have shown a strong correlation between TB treatment history and H resistance [3].

Previous systematic meta-analyses have shown that H resistance reduces the likelihood of treatment success and increases the risk of developing resistance to other important first-line drugs, such as R, thereby increasing the risk of multidrug-resistant TB (MDR-TB) [4,5]. Prior studies have also demonstrated that Hr-TB patients receiving only first-line regimens may experience adverse outcomes, compared to drug-sensitive TB patients [5,6]. However, the optimal regimen and duration of treatment for Hr-TB have not been fully established yet and the recommended treatment regimens vary according to guidelines [7]. In addition, various personalized treatment regimens are widely used in clinical practice without sufficient clinical evidence [8–10].

Currently, the World Health Organization (WHO) [2] recommends six-month (H) + R + ethambutol (E) + pyrazinamide (Z) + fluoroquinolone (FQ) treatment for Hr-TB, particularly in patients with disseminated disease. However, these recommendations are mostly based on observational studies and, therefore, further studies are needed to optimize the treatment of Hr-TB. The aim of this study was to evaluate the treatment regimens and results of H-resistant pulmonary TB patients.

## 2. Material and method

### 2.1. Study design and study population

This single-center, retrospective study was conducted at Yedikule Chest Diseases and Thoracic Surgery Training and Research Hospital, Department of Chest Diseases Tuberculosis Clinic between January 2015 and December 2017. Medical records of 213 consecutive patients with Hr-TB were reviewed. Among them, a total of 25 patients were excluded from the analyses because of the following reasons: a TB patient who did not start treatment or whose treatment was interrupted for 2 consecutive months or more (n = 19), patients that were transferred out to another treatment unit (n = 3), death from disease progression before the treatment initiation (n = 3). The remaining 188 patients represented the final study population. Data including demographic characteristics of the patients, comorbidities, test results for human immunodeficiency virus (HIV) infection, sputum smear and cultures, concomitant drug resistance patterns, TB treatment history, chest radiographs, presence of cavity on chest radiographs, treatment regimens applied, and related side effects, and treatment outcomes were recorded. Written informed consent was obtained from each patient for all diagnostic and therapeutic procedures. The study protocol was approved by the Institutional Ethics Committee (No: 2021-112, Date: 08.04.2021) and conducted in accordance with the principles of the Declaration of Helsinki.

### 2.2. Identification of isolates and drug resistance studies

All samples were subjected to the homogenization-decontamination process for TB examination. They were taken into Löwenstein-Jensen (LJ) medium and Mycobacterium Growth Indicator Tube (MGIT) 960 (Becton Dickinson Diagnostic Instruments, MD, USA) tubes and left for incubation. The positivity was confirmed by microscopic smear for acid-fast bacilli (AFB) examination from all samples with growth in both LJ and MGIT 960 systems. Susceptibility tests were performed for H, R, E, and streptomycin (S) in isolates of the isolated M. tuberculosis complex. Sensitivity tests were performed according to international standards and the MGIT 960 system was used. Final critical concentrations were 0.1 μg/mL for H, 1.0 μg/mL for R, 5.0 μg/mL for E, and 2.0 μg/mL for S^3^.

Resistance to H was defined as phenotypic resistance to alone H or to S with H without resistance to other first-line anti-TB drugs [11]. In our center, a genotypic drug susceptibility test is available since 2017, which is one of the four referral hospitals following TB patients across the country. As of this date, a genotypic drug susceptibility test for R and H is performed from the sputum sample of every smear-positive patient, regardless of whether the patient is at the risk group for drug resistance. If MDR-TB or H resistance is identified and quinolone is to be added to the treatment, quinolone resistance and resistance for injectable agents are evaluated. Therefore, only 49 patients among the patients included in the study were able to be diagnosed with H resistance at the beginning of the treatment with the genotypic drug susceptibility test.

### 2.3. Definitions of treatment outcomes

Treatment outcomes were defined according to the WHO Report of Definitions and reporting framework for tuberculosis 2013^4^. Treatment outcomes are definitions for both pulmonary and extrapulmonary TB disease including (1) treatment success (a-cure and b- treatment complete), (2) treatment failure, (3) lost to follow-up, and (4) death. Only cure definition is related to AFB smear or culture-positive pulmonary TB cases. The patient whose culture was negative in the last month of the treatment and also negative at least once in the previous period was accepted as the only cure. Treatment complete was defined as the completion of treatment without evidence of failure, but not meeting the cure criteria.

Treatment failure was defined as having consistently positive sputum culture results at and after five months of treatment. Relapse was defined as a recurrence of pulmonary TB after initially successful treatment. Additional drug resistance development was investigated in the patients with relapse. Mortality was defined as death for any reason before the initiation of the treatment or during the treatment.

If two consecutive cultures were found to be negative, the first culture-negative date was recorded as the culture-negative time. The patients who did not start treatment or whose treatment was interrupted for two consecutive months or longer were evaluated as, lost to follow-up patients. Sputum culture conversion was defined as a negative culture in at least two consecutive sputum samples without a subsequent positive culture. Time to culture negativity was determined in the patients receiving and not receiving FQ based on monthly sputum sample analysis. The effect of the FQ-containing treatment regimen on culture conversion time was evaluated. Relapse was defined as a recurrence of pulmonary TB after initially successful treatment. The long-term results of the patients treated for a minimum of three and a maximum of five years were assessed. Treatment success without evidence of relapse was defined as a positive result during the follow-up period, while negative results were treatment failure, relapse, and death.

### 2.4. Treatment setting and protocol

**Figure 1:**
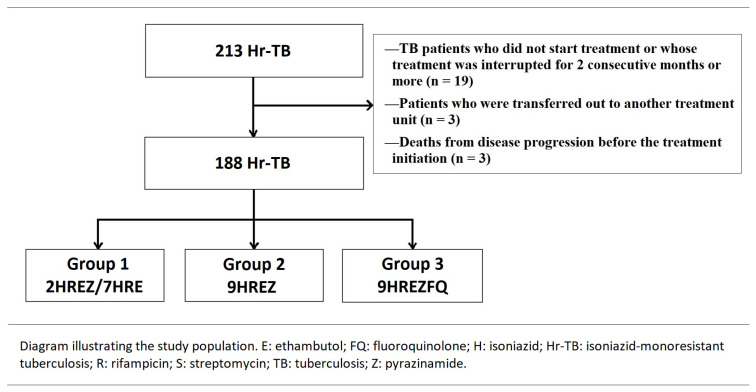
Study population.

To the Republic of Turkey, Ministry of Health, National TB Control Program guidelines [12], three sputum samples were taken from each patient at the time of diagnosis, once a month during treatment. Although the patients were followed regularly every month, some of the patients could not provide sputum analysis every month during follow-up, as they were unable to produce sputum. The Directly Observed Treatment (DOT) Strategy was implemented by healthcare professionals to ensure that available treatments were taken timely throughout the treatment period. Comorbid diseases of the patients and the drugs they used were recorded. The treatment regimens of the patients were determined and divided into three groups: Intensive phase of 2 months of HRZE followed by a continuation phase of 7 months of HRE (Group 1; 2HREZ/7HRE), HREZ for nine months (Group 2; 9HREZ); and HRZE + FQ for nine months (Group 3; 9HREZFQ) (Figure 1). The effect of FQ on treatment outcomes was further evaluated by creating groups receiving FQ (Group 3) and those not receiving FQ (Group 1 and Group 2). The tuberculosis dispensary was contacted for the treatment and results of all patients.

### 2.5. Statistical analysis

Statistical analysis was performed using the SPSS version 24.0 software (IBM Corp., Armonk, NY, USA). Normality distribution was checked using graphical (histograms, probability curves) and numerical methods (Kolmogorov-Smirnov and Shapiro-Wilk tests). Continuous variables were expressed in mean ± standard deviation (SD) or median (interquartile range [IQR]), while categorical variables were expressed in number and frequency. The analysis of variance (ANOVA) test was used to compare continuous variables. Categorical variables were analyzed using the chi-square and Fisher’s exact tests. The probability curves of patients who remained culture positive for M.tb were analyzed using the Kaplan-Meier method and compared with the log-rank test. A two-sided p-value of < 0.05 was considered statistically significant.

## 3. Results

Of 188 Hr pulmonary TB patients included in the study, 123 (65.4%) were males and 65 (34.6%) were females with a mean age of 40.1 ± 16.2 years. The most common comorbidity was diabetes mellitus (DM) in 14 patients (7.4%), followed by hypertension (3.2%). All patients were negative for HIV infection. Thirty-five patients (18.6%) had a history of previous TB treatment. Only H resistance was found in 119 patients (63.3%), while 69 (36.7%) patients had S resistance combined with H resistance. The diagnosis was made based on sputum examination in 160 patients (85%) and bronchoalveolar lavage fluid samples in 28 patients (15%). In 94 (50%) of the patients, bacillus was observed bacillus negative sputum smears and the diagnosis was made with culture positivity. A total of 143 patients (76%) were under follow-up in the outpatient setting, while the treatment of 45 patients (24%) was initiated in the in-hospital setting in the TB ward.

Since molecular testing has been performed in our center since 2017, only 49 patients (26.1%) underwent molecular testing. While promoter resistance was detected in 31 (16.5%) of these patients, katG deletion was detected in 18 patients (9.6%). Molecular tests could not be performed on the patients in the initial phase. Since the level of INH resistance could not be determined, INH treatment was continued.

Treatment-related side effects were observed in 33 patients (17.5%). Twenty-five (13.2%) patients had an asymptomatic elevation in liver enzymes, which settle with continued use of the drug that did not require a treatment switch, five patients had visual impairment due to E, and three patients had allergic skin reactions controlled with antihistamine drugs.

Seventy-seven patients in Group 1 received an intensive phase of 2 months of HRZE followed by a continuation phase of 7 months of HRE (Group 1; 2HREZ/7HRE), 78 patients in Group 2 received HREZ for nine months, and 33 patients in Group 3 received HREZFQ for nine months.

The demographic characteristics of 188 patients grouped according to treatment regimens are summarized in Table 1.

For all patients, INH treatment was continued because the level of INH resistance could not be determined. Treatment doses were initiated according to the WHO recommendations and national guidelines as suitable to the weight, and moxifloxacin (Mfx) 400 mg daily was prescribed in the treatment regimen containing FQ.

There was no statistically significant difference in comorbidities, DM rate, presence of cavitary lesions on chest radiographs, and extent of the disease between the patients with positive and negative results (p > 0.05).

However, in the patients with negative results, the rate of AFB positivity in sputum smear samples taken at the beginning of treatment (p = 0.010) and previous TB treatment history was found to be statistically significantly higher (p = 0.020) (Table 2).

Totally, 174 (92.6%) of 188 patients with Hr pulmonary TB achieved treatment success. The cure was observed in 97 patients (51.6%) and treatment completed was achieved in 77 patients (41.0%). Five patients (2.7%) had treatment failure. In four (2.1%) of 174 patients with treatment success, relapse was seen during the one-year follow-up period. Eighteen patients (9.6%) had unfavorable outcomes, including treatment failure in five (2.7%), death during follow-up in nine (4.8%), and relapse after the initial treatment success in four patients (2.1%). Treatment outcomes are summarized in Table 3.

**Figure 2:**
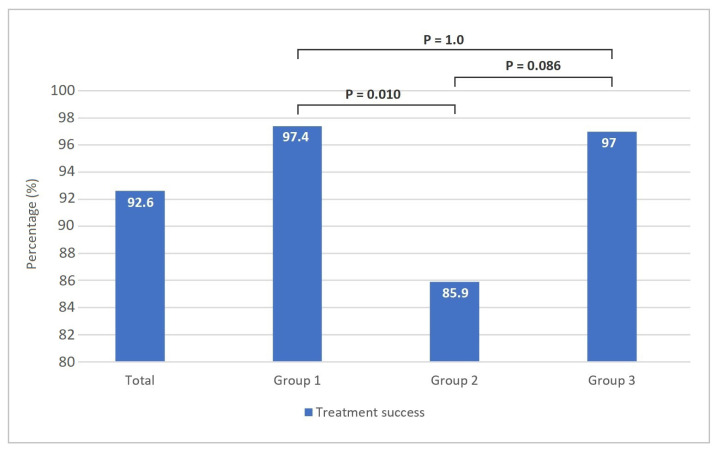
Treatment regimens and treatment success.

**Figure 3:**
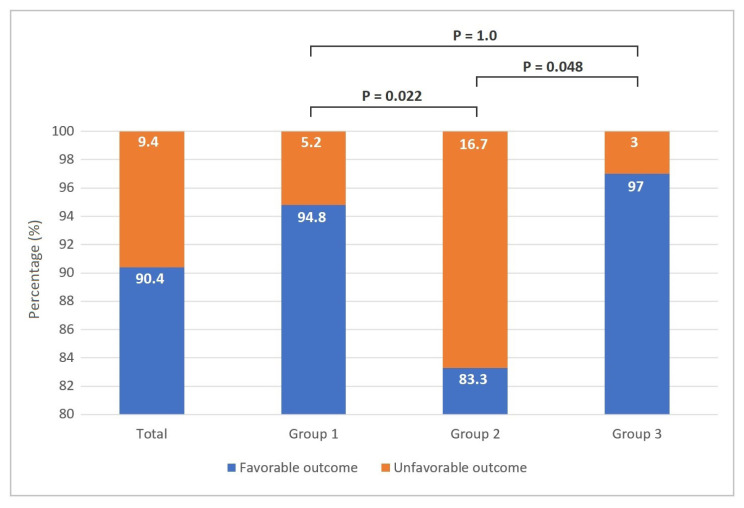
Treatment regimens and favorable outcomes.

The patients receiving the FQ-containing treatment regimen (Group 3) had numerically higher treatment success rates than those treated with HREZ for nine months (Group 2) (97.0% vs. 85.9%, p = 0.086), but did not reach significance due to a small sample size. The treatment success rate was found to be statistically higher in group 1 compared with those in group 2 (97.4% vs. 85.9%; p = 0.010) (Figure 2). Group 3 and group 1 had statistically significant favorable outcomes, compared to group 2 (group 2 vs. group 3, p = 0.048; group 1 vs. group 2, p = 0.022) (Figure 3).

After the initial treatment success, none of the patients receiving FQ had relapses. However, this difference did not reach statistical significance due to the low sample size (p = 0.647). In all relapse cases, MDR-TB developed.

Totally, MDR-TB developed in 10 (5.3%) patients, while it did not develop in the patients receiving the FQ regimen. Drug resistance acquired in these ten patients included was acquired resistance to R (n = 10) and E (n = 4). Although MDR-TB development was observed in the groups that did not receive FQ, this difference was not statistically significant (p = 0.256). During the treatment, mortality was observed in nine patients (4.8%). Eight of these patients in Group 2 died during treatment.

It was thought that mortality was more common in group 2 patients, as the clinician continued the treatment with HRZE for 6–9 months without reducing the medication due to bilateral cavitary disease and delayed culture conversion.

For the same reason, it was thought that the treatment success of the patients in Group 1 was higher than the patients in Group 2.

**Figure 4:**
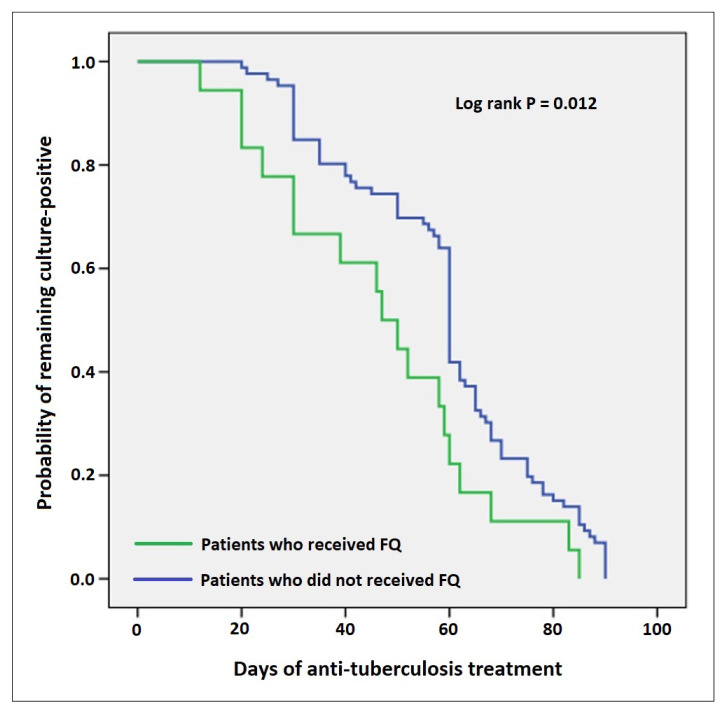
Probability curves of patients who remained culture-positive for Mycobacterium tuberculosis in the two groups using the Kaplan-Meier method. FQ: fluoroquinolone.

In our study, we also investigated the effect of additional Mfx to the standard anti-TB regimen on the duration of sputum culture conversion, and regimens with and without FQ were compared. In both groups, time to culture negativity was calculated for the patients in whom monthly sputum samples could be obtained. The median time to culture negativity was 46.9 ± 21.3 days and 58.9 ± 19.0 days in the groups with and without FQ, respectively. A statistically significant difference was observed between the two groups in terms of the period until the culture conversion (p = 0.019). The time to culture conversion was shorter in the Mfx group. In addition, the probability curves of the patients who remained culture-positive for M.tb in the patient groups with and without FQ were analyzed using the Kaplan-Meier method. In patients who received FQ, the number of days remaining as culture-positive was shorter than in the other groups. Additional FQ to the treatment regimen of the patients shortened the time to culture negativity and decreased the number of culture-positive days (Figure 4).

## 4. Discussion

In the present study, we investigated the treatment regimens and results of H-resistant pulmonary TB patients. The results of this study showed that the addition of FQ to treatment in Hr-TB prevented acquired drug resistance. Widespread disease and delayed culture conversion were found to be associated with treatment failure, and the addition of FQ to the treatment regimen accelerated culture conversion. For this reason, we think that adding FQ to the treatment regimen will increase the success of treatment and prevent acquired drug resistance, especially in severe diseases. However, the contribution of Z given throughout the treatment to the success of the treatment has not been demonstrated. Administration of Z was deemed unnecessary throughout the treatment due to the increased risk of toxicity and impaired adherence to treatment.

In a study, Stagg et al. [13] evaluated the results of 594 Hr-TB patients, of whom 330 (55.6%) were treated with HRZE and 211 (35.5%) with HRZEFQ. The median overall treatment duration was 11.9 months and the median Z treatment duration was 2.1 months. In a univariate logistic regression model comparing the RZE strategy predominantly with Mfx and without FQ, the authors found no significant difference in the probability of negative overall outcomes between the two regimens (odds ratio [OR] 1.05, 95% confidence interval [CI] 0.60–1.82). The aforementioned authors also showed that if the duration of HRZE treatment was long enough (12 months), Z might not need an FQ, even if it had relatively short durations (median 2 months at baseline). Despite its inherent limitations such as its retrospective nature and lack of a randomized-controlled design, the authors suggested that the 12-month regimen without an FQ could result in fewer adverse events by the WHO recommendations, although they did not report the type and frequency of events that occurred in their cohorts.

Although studies conducted in the 1970s and 1980s showed high success rates in Hr-TB patients receiving regimens consisting of first-line drugs, the relapse rate was higher in these patients, compared to drug-sensitive TB patients [14]. A large-scale retrospective cohort study showed that all types of H resistance were associated with treatment failure in both new and retreatment cases [15]. In another study, H resistance was associated with treatment failure, resulting in acquired drug resistance in a significant proportion of cases, including those of MDR-TB [16].

In a meta-analysis including 57 randomized controlled trials conducted by Menzies et al. [4], the rates of treatment failure associated with H resistance were 10.9 times higher, relapse rates 1.8 times higher, and acquired drug resistance rates 5.1 times higher than fully susceptible TB cases. In several studies, positive treatment outcomes of Hr-TB patients are approximately 80% to 90% [9,17,18]. Recent studies have suggested that FQ may be useful in the treatment of Hr-TB [10,17].

Resistance to first-line drugs to treat TB is an important public health issue that hinders the WHO End TB strategy^5^. According to the most recent estimates from 149 countries in the 2003–2017 period, H resistance without concurrent R resistance is still present in 7.1% of new TB cases and 7.9% of previously treated TB patients. In response to the general growing problem of drug-resistant TB, the WHO published consolidated guidelines on current evidence for the treatment of H resistance in 2018.

Data sets of only 251 patients from 15 observational studies including fluoroquinolones (FQs) as a part of standardized TB regimens for at least one month in addition to REZ for at least six months which was primarily designed for Hr-TB were compared with 1350 patients not receiving FQ regiments. A total of 251 patients who received FQs were found to be significantly more likely to be treated. There was no significant difference between the two groups in terms of mortality. With six-month HREZFQ regimens, the median duration of FQ, R, E, and Z use was 6.1 months, 9.0 months, 9.0 months, and 8.9 months, respectively. Based on the meta-analysis data, the WHO recommended limited use of levofloxacin (Lfx) at a dose of 750 to 1000 mg for at least six months, particularly as a part of a REZ regimen (with or without H) for Hr-TB patients^6^. In this meta-analysis of Fregonese et al. [19], the six-month REZ regimen provided favorable results in the patients with Hr-TB, and treatment longer than six months was not associated with improved results. These findings provide evidence of the benefit of the addition of FQ to a core regimen containing REZ, although the optimum duration and specific FQ type have not been clarified yet. An additional implication is that the standardized retreatment regimen appears to offer limited benefit in patients with confirmed Hr-TB [19].

Considering the FQ selection in the regimes, despite the WHO recommendation on the use of Lfx, we used Mfx in our study. Although we could not comment on the choice of the most optimal FQ, as all patients treated with FQ-containing regimens were prescribed Mfx, we achieved significantly successful results with our choice of Mfx. In a study, Kang et al. [20] showed that Lfx and Mfx preferences for the treatment of MDR-TB patients did not affect the treatment outcomes. In a recent study evaluating the therapeutic value of FQs in TB treatment, the inclusion of expanded Pharmacokinetic/Pharmacodynamic *(*PK*/*PD*)* analysis in the armamentarium of drug development tools was found to be necessary to elucidate the role of Mfx, gatifloxacin, and Lfx in TB treatment using the correct dosage [21]. The duration of treatment in INH-resistant patients has been 9 months according to the Turkish National Guideline since 2003 [12]. The effect of differences in treatment regimen durations on treatment outcomes may be clarified by future studies.

Furthermore, the drug susceptibility test results of the FQs are critical while deciding on the selection of FQ in the treatment of Hr-TB. Another limitation of our study is that the resistance status for Z and FQ is not known when deciding on the treatment regimens of the patients.

Since molecular testing has been performed in our center since 2017, only 49 patients (26.1%) underwent molecular testing. Response to treatment may vary according to genotypic forms of H resistance and, therefore, complete genotypic information would provide more information in future studies. In addition, planning the treatment according to the genotypic forms of H resistance and deciding to add H to the treatment regimen may provide additional benefits to the patients.

The main limitation of the present work is that it is a retrospective one, it has an observational nature and even the sample sizes of the groups are small to achieve statistical power. Maybe in the future prospective and randomized studies will support this hypothesis.

In conclusion, the addition of FQ to the core regimen for the treatment of H-resistant pulmonary TB, particularly in common and cavitary diseases, may contribute to the success of the treatment and to the prevention of the development of acquired drug-resistant disease with relapse. However, the administration of Z during the treatment does not seem to contribute to the success of the treatment. Since Z is also associated with an increased risk of toxicity and reduced treatment compliance, the benefit of this treatment during the treatment period is unclear. Based on these findings, we suggest that additional FQ to standard treatment regimens are associated with more favorable results in Hr-TB and should be considered for the treatment of Hr pulmonary TB. Nonetheless, given the worldwide burden of Hr-TB, further large-scale, randomized-controlled studies are needed including these specific patient populations and evaluating novel therapeutic regimens, and investigating the efficacy, safety, and tolerability of pyrazinamide in the short- and long-term.

## Figures and Tables

**Table 1:** Demographic and clinical characteristics of patients.

Parameters	Total (N = 188)	Group 1 (N = 77)	Group 2 (N = 78)	Group 3 (N = 33)	P-value
**Age, years**	40.1 ± 16.2	40.1 ± 16.9	40.1 ± 16.5	40.2 ± 14.5	1.0
**Sex (male)**	123 (65.4)	49 (63.6)	52 (66.7)	22 (66.7)	0.912
**Diabetes mellitus**	14 (7.4)	3 (3.9)	6 (7.7)	5 (15.2)	0.119
**Hypertension**	6 (3.2)	1 (1.3)	3 (3.8)	2 (6.1)	0.391
**COPD**	1 (0.5)	0 (0.0)	1 (1.3)	0 (0.0)	0.492
**Asthma**	2 (1.1)	0 (0.0)	1 (1.3)	1 (3.0)	0.354
**Chronic kidney disease**	2 (1.1)	0 (0.0)	2 (2.6)	0 (0.0)	0.240
**Coronary artery disease**	3 (1.6)	1 (1.3)	2 (2.6)	0 (0.0)	0.593
**Heart failure**	4 (2.1)	1 (1.3)	3 (3.8)	0 (0.0)	0.354
**Previous stroke**	1 (0.5)	0 (0.0)	1 (1.3)	0 (0.0)	0.492
**Malignancy**	2 (1.1)	1 (1.3)	1 (1.3)	0 (0.0)	0.804
**Hypothyroidism**	4 (2.1)	2 (2.6)	0 (0.0)	2 (6.1)	0.121
**Silicosis**	1 (0.5)	1 (1.3)	0 (0.0)	0 (0.0)	0.485
**Positive sputum AFB smear**	94 (50.0)	38 (48.4)	46 (59.0)	10 (30.3)	0.022
**Cavitary lesions in chest radiography**	117 (62.6)	41 (53.9)	54 (69.2)	22 (66.7)	0.127
**Lesion in chest radiography**					
**Bilateral**	80 (42.6)	28 (36.4)	39 (50.0)	13 (39.4)	0.211
**Unilateral**	108 (57.4)	49 (63.6)	39 (50.0)	20 (60.6)	

Values are mean ± SD or n (%)

Abbreviations: AFB, acid-fast bacillus; COPD, chronic obstructive pulmonary disease

**Table 2:** Comparison of clinical characteristics of patients with favorable versus unfavorable outcomes.

Characteristic	Total (N = 188)	Favorable outcomes (N = 170)	Unfavorable outcomes (N = 18)	P-value
**Positive sputum smear test**	94 (50.0)	80 (47.1)	14 (77.8)	0.01
**Diabetes mellitus**	14 (7.4)	14 (8.8)	0 (0.0)	0.368
**Hypertension**	6 (3.2)	6 (3.5)	0 (0.0)	1.0
**Coronary artery disease**	3 (1.6)	2 (1.2)	1 (5.6)	0.262
**Heart failure**	4 (2.1)	4 (2.4)	0 (0.0)	1.0
**Previous history of TB treatment**	35 (18.6)	28 (16.5)	7 (38.9)	0.020
**Cavitary lesions in chest radiography**	117 (62.6)	103 (60.9)	14 (77.8)	0.161
**Lesion in chest radiography**				
**Bilateral**	80 (42.6)	72 (42.4)	8 (44.4)	0.864
**Unilateral**	108 (57.4)	98 (57.6)	10 (55.6)	

Data are presented as numbers (%).

**Table 3:** Treatment regimens and clinical outcomes.

Outcomes	Total (N = 188)	Group 1 (N = 77)	Group 2 (N = 78)	Group 3 (N = 33)	P-value
**Treatment success**	174 (92.6)	75 (97.4)	67 (85.9)	32 (97.0)	0.014
**Cured**	97 (51.6)	36 (46.8)	43 (55.1)	18 (54.5)	0.541
**Treatment completed**	77 (41.0)	39 (50.6)	24 (30.8)	14 (42.4)	0.041
**Treatment failure**	5 (2.7)	2 (2.6)	3 (3.8)	0 (0.0)	0.341
**Death**	9 (4.8)	0 (0.0)	8 (10.3)	1 (3.0)	0.010
**MDR TB**	10 (5.3)	4 (5.2)	6 (7.7)	0 (0.0)	0.256
**Relapse after treatment success**	4 (2.1)	2 (0.0)	2 (2.6)	0 (0.0)	0.647
**Favorable outcome**	170 (90.4)	73 (94.8)	65 (83.3)	32 (97.0)	0.020
**Unfavorable outcome**	18 (9.6)	4 (5.2)	13 (16.7)	1 (3.0)	

Data are presented as numbers (%).

Abbreviations: MDR TB; Multidrug resistance tuberculosis

## Footnotes

1 World Health Organization‎ (2019). Global tuberculosis report 2019. World Health Organization [online]. Website https://apps.who.int/iris/handle/10665/329368 [accessed 17 October 2019].

2 World Health Organization (‎2018)‎. WHO treatment guidelines for isoniazid-resistant tuberculosis: supplement to the WHO treatment guidelines for drug-resistant tuberculosis. World Health Organization [online]. Website https://apps.who.int/iris/handle/10665/260494 [accessed 15 March 2018].

3 World Health Organization. (‎2018)‎. Technical manual for drug susceptibility testing of medicines used in the treatment of tuberculosis. World Health Organization [online]. Website https://apps.who.int/iris/handle/10665/275469 [accessed 23 October 2018].

4 World Health Organization. (‎2013)‎. Definitions and reporting framework for tuberculosis – 2013 revision: updated December 2014 and January 2020. World Health Organization [online]. Website https://apps.who.int/iris/handle/10665/79199 [accessed 9 April 2013].

5 World Health Organization (WHO). End TB Strategy [online]. Website https://www.who.int/publications/i/item/WHO-HTM-TB-2015.19 [accessed 16 August 2015].

6 World Health Organization (‎2018)‎. WHO treatment guidelines for isoniazid-resistant tuberculosis: supplement to the WHO treatment guidelines for drug-resistant tuberculosis. World Health Organization [online]. Website https://apps.who.int/iris/handle/10665/260494 [accessed 15 March 2018].
